# Attribute Selection Hybrid Network Model for risk factors analysis of postpartum depression using Social media

**DOI:** 10.1186/s40708-023-00206-7

**Published:** 2023-10-31

**Authors:** Abinaya Gopalakrishnan, Raj Gururajan, Revathi Venkataraman, Xujuan Zhou, Ka Chan Ching, Arul Saravanan, Maitrayee Sen

**Affiliations:** 1 School of Business, University of Southern Queensland, Springfield, QL Australia; 2 Department of Networking and Communications, School of Computing, SRM Institute of Science and Technology, Chennai, India; 3 Department of Psychiatry, SRM Medical College Hospital & Research Centre, Chennai, India; 4 Department of Obstetrics and Gynaecology, SRM Medical College Hospital & Research Centre, Chennai, India

**Keywords:** Postpartum depression, Social media, Physiological questionnaire, Attribute selection, Neural networks

## Abstract

**Background and objective:**

Postpartum Depression (PPD) is a frequently ignored birth-related consequence. Social network analysis can be used to address this issue because social media network serves as a platform for their users to communicate with their friends and share their opinions, photos, and videos, which reflect their moods, feelings, and sentiments. In this work, the depression of delivered mothers is identified using the PPD score and segregated into control and depressed groups. Recently, to detect depression, deep learning methods have played a vital role. However, these methods still do not clarify why some people have been identified as depressed.

**Methods:**

We have developed Attribute Selection Hybrid Network (ASHN) to detect the postpartum depression diagnoses framework. Later analysis of the post of mothers who have been confirmed with the score calculated by the experts of the field using physiological questionnaire score. The model works on the analysis of the attributes of the negative Facebook posts for Depressed user Diagnosis, which is a large general forum. This framework explains the process of analyzing posts containing Sentiment, depressive symptoms, and reflective thinking and suggests psycho-linguistic and stylistic attributes of depression in posts.

**Results:**

The experimental results show that ASHN works well and is easy to understand. Here, four attribute networks based on psychological studies were used to analyze the different parts of posts by depressed users. The results of the experiments show the extraction of psycho-linguistic markers-based attributes, the recording of assessment metrics including Precision, Recall and F1 score and visualization of those attributes were used title-wise as well as words wise and compared with daily life, depression and postpartum depressed people using Word cloud. Furthermore, a comparison to a reference with Baseline and ASHN model was carried out.

**Conclusions:**

Attribute Selection Hybrid Network (ASHN) mimics the importance of attributes in social media posts to predict depressed mothers. Those mothers were anticipated to be depressed by answering a questionnaire designed by domain experts with prior knowledge of depression. This work will help researchers look at social media posts to find useful evidence for other depressive symptoms.

## Highlights

1. A novel hybrid attribute selection model has been proposed for the prediction of Post-Partum Depression.
2. Attribute Hybrid Networks have been tested on a unique dataset that includes both the PDSS questionnaire and social media posts of the recruited individuals.
3. The model applies a depression theory to select each attribute using interconnected neural networks and a post-level attention layer.
4. The experimental results demonstrate that the Attribute Selection Hybrid Network outperforms other baseline models.
5. The study employs title- and word-wise word clouds visualization to compare daily life, depression, and postpartum depression.
6. The proposed Attribute Selection Hybrid Networks model is also capable of predicting various mood disorders.

## Introduction

Postpartum depression (PPD) is a type of depression that affects women after giving birth. According to the World Health Organization’s 10th revision of the International Statistical Classification of Diseases and Related Health Problems (2009), PPD is a ”behavioural and psychological problem” that occurs within the first six weeks after childbirth. PPD is more common in women than men [1], and it can have a range of emotional symptoms, such as crying, worry, sadness, sleep problems, confusion, and irritability. PPD is associated with suicidal thoughts and usually requires specialized treatment. However, a more severe form of PPD called postpartum psychosis can occur in a small percentage of women (0.1$-$0.2%) and is characterized by symptoms such as restlessness, sleep disturbances, paranoia, disordered thinking, impulsivity, hallucinations, anxiety, and delusions. Postpartum psychosis is a severe condition that requires immediate treatment and can be especially common in mothers who are 35 years old or older. It typically reaches its peak in the first two weeks after delivery.

There is growing recognition among professionals that postpartum depression (PPD) has significant impacts on a mother’s relationships with her family, spouse, and baby, as well as on the mother-infant connection and the long-term emotional and cognitive development of the child [2–5]. PPD is associated with a poor quality of life and affects the language used in social media activities [6]. Many studies have attempted to identify depressed individuals by analyzing language use in social media, focusing on differences in word usage between depressed and non-depressed groups [7–12]. Some studies have tried to predict depression by comparing subjects with depression to control groups [13, 14]. In contrast, others have used sentiment analysis techniques based on the idea that people with depression are more likely to express negative emotions. However, previous research has often relied on small datasets and has not effectively explained the detection results with crucial concepts in the field. A few studies have used neural network approaches [15–17], but still, more in-depth research is needed on subsequent steps such as diagnosis and prevention of PPD.

According to the findings of Hoyun et al. [18], and Eichstaedt and colleagues [19], individuals may post about their depression and therapy on social media, and the language used on Facebook can accurately predict depression based on medical records. De Choudhury and colleagues [8, 9] developed a statistical model to predict extreme postnatal behavioural changes based on linguistic and emotional correlations for postnatal changes in new mothers. Reece and his colleagues [20] also built computational models to predict the likelihood of post-traumatic stress disorder in Twitter users. These studies demonstrate the potential of social media as a source of signals for predicting present or future episodes of depression.

This research aims to investigate the attributes associated with the worsening of PPD to facilitate the development of new methods for identifying at-risk mothers and provide direction for effective therapies. It provides a digital safety net framework to support new mothers during a significant life transition. It builds on previous research that has found a link between depression and specific linguistic characteristics. It aims to expand the scope of social media-based mental health measures by creating a framework that recognizes text-based signs of Postpartum depression as similar [21]. This work can be beneficial in identifying at-risk mothers early and offering them support on time.

Highlights of the novel contributions are listed as follow:

- This study combines active as well as passive monitoring for predicting the postpartum depression.
- To predict the postpartum depression hybrid attribute networks are used to predict various form of attributes which expressed in the posts of young mothers accurately
- A novel Post level attention mechanism are used to choose and process depressed postings among other posts.
- Word cloud based comparison is also carried out to confirm truth worthiness of the depressed posts.

The remainder of the paper is structured as follows: Sect. 2 briefly discusses the related works. Section 3 introduces the datasets used in the experiments, including the PPDS questionnaire and Facebook data from depressed mothers. Section 4 describes the proposed Attribute Selection Hybrid Network Models architecture. Section 5 presents word cloud results and the proposed architecture for depression analysis. Section 6 discusses the results, and the conclusion is presented in Sect. 7.

## Related work

Various studies are being conducted to gain new insight into diagnosing PDD depression by analyzing the association between mental health and language usage [22]. Studies on depression and other mental health illnesses have become more challenging as social media and the Internet have evolved. Online platforms such as Facebook, Twitter, and Reddit provide a new opportunity for innovative research by offering a vast amount of text data and social information that can be used to understand women’s behavioural tendencies. Machine learning (ML) and deep learning (DL) techniques have been used to analyze textual data and investigate the impact of social networks on users’ mental health. Existing research has been analyzed from multiple perspectives, including text and framework levels.

### New mothers depression detection in social media

According to polls conducted by Nielsen Wire in 2012, 72% of mothers use Facebook compared to other social media platforms to express their feelings [23]. Over the past several years, several types of research have been carried out to explore the social media usage of women who have recently given birth. These studies have focused on blogging [24], pregnancy and motherhood forums [24, 25], and Facebook. McDaniel and colleagues [24] found that the frequency of posts from new mothers was related to their feelings of interpersonal connectedness to extended family and friends, as well as to express their feelings regarding social support and maternal welfare. Gibson and Hanson [26] found that new mothers saw Facebook as a valuable platform for creating a new identity, maintaining social connections after giving birth, and finding information and comfort about their decisions and worries about raising a baby. These findings are based on ethnographic studies. According to Schoenebeck [27], the posts on the anonymous message board YouBeMom.com define new social norms and expectations that shape the culture of online mothers.

All those previous studies suggest that online social technologies provide new mothers with opportunities to use their social networks and to discover a liberated outlet for conversing, venting, and exchanging parenting information with other new mothers. This study continues to explore streams of online social activity to understand better the role played by online social groups in supporting PPD and the absence of such support.

In related research, De Choudhury et al. analyzed tweets from new mothers to discover [28] and forecast [29] significant behavioural changes postpartum. Instead of accessing actual data on PPD results, the investigations relied on identifying substantial changes made on Twitter. To the best of our knowledge, this report is the first study to reveal postpartum depression predictions based on new mothers’ usage of Facebook in conjunction with PDD scores.

### Various framework for processing social media data

Numerous studies have been undertaken to explain the classification findings from neural networks to examine the essential attributes contributing to performance and to strengthen it further [30, 31]. Various vision-related investigations have employed neural visualization with representations learned from succeeding layers to provide human-interpretable data [32]. Several researchers have applied interpretable approaches to natural language processing, concentrating primarily on interpreting vector-based models for various applications. In contrast to analyzing input patterns to analyze activated internal neurons, Palangi et al. [33] interpreted lexical-semantic meanings and grammatical functions for each word based on internal representation. However, the inability to provide a detailed explanation is a drawback of interpreting a result by studying attention or neurons. Kshirsagar et al. [34] made an effort to generate explanations about detected results for suicidal posts by using representation learning. However, they conducted the attention mechanism only on the words in a post, which has limitations. Applying the attention mechanism to a mother’s posts proved to be a difficult task since the proportion of posts containing depression indicators was insufficient, meaning that the majority of the new mother’s posts did not have information that was sufficiently helpful for depression detection. To interpret the attribute representations related to various depression factors learned from hybrid attribute networks to understand which attributes are activated significantly during depression detection. To do this, the concepts discussed previously were utilized.

### Summary of research gaps

Predicting PPD has not been widely researched in the past, likely due to the challenges associated with collecting longitudinal data on mothers’ behaviour over a long period. Traditional methods, such as observation and in-person interviews, can be expensive and intrusive, making it challenging to collect enough data to draw meaningful conclusions. These approaches are mainly based on labour-intensive methods of manually gathering the attributes, which show limited performance for classification. Lack of domain knowledge about the attributes plays a vital role in predicting PPD through the posts shared through social media. The advent of online social platforms like Facebook has opened up new opportunities for research in this area. These techniques, however, still don’t give a clear explanation for why certain newly delivered mothers have been labelled as depressed.

## Materials

This section provides an introduction to the dataset used for this study’s experiments. It includes a description of the dataset’s primary characteristics, the corresponding task, and the criteria used for evaluating it.

### Ethical clearance

The data collection for this study was approved by the Institutional Ethical Committee (IEC) at SRM Medical College and Research Center (SRMC &RC) in Chennai, India. Data was collected in 2022 from mid-April to mid-July. Each participant signed a consent form indicating that she had read and understood the terms and conditions of the study. All data collection and analysis were conducted under the applicable ethical guidelines and regulations.

### Participants selection

Clinicians identified a potential participant pool for the study, and data collection was done efficiently. Using a sequential participant selection method, mothers who had given birth at SRMC &RC in Chennai, India and came for post-checkups within six weeks of delivery were included in the study. This ethical clearance allowed for collecting data from mothers at a critical time postpartum, increasing the chances of identifying postpartum depression.

#### Inclusion criteria

These individuals were informed about the objective of the study and voluntarily agreed to participate without any pressure or reservation based on the following criteria:

- Mothers between the ages of 19 and 35 who had given birth.
- Participants were able to read and comprehend the study’s details and complete the consent form mentally.
- The type of delivery (spontaneous or induced) does not matter; mothers can be primigravida or multigravida.

These criteria were used to ensure that the study sample is representative of the population of mothers who have given birth and are within a specific age range. Additionally, including mothers with different types of deliveries and parity increases the findings’ generalizability.

#### Exclusion criteria

Individuals were not eligible to participate in the study based on the following criteria:

- Mothers with multiple fetal pregnancies.
- Mothers who are convinced through IVF treatments.
- Mothers with a complicated obstetric history.
- Mothers considered high-risk pregnancies, such as those with gestational diabetes mellitus, preeclampsia, chronic disease, and fetal anomalies.

These criteria were used to exclude certain groups of mothers at a higher risk of postpartum depression and whose experiences may not represent the overall population of mothers who have given birth. Additionally, these groups of mothers may have different medical needs and be unable to participate fully in the study.

### Dataset collection

#### Postpartum Depression Screening Scale PDSS survey

Data were collected from volunteers who had given birth at SRM Medical College and Research Center and participated in psychological research. Participants completed the Postpartum Depression Screening Scale (PDSS), a questionnaire that helps determine a depression score on a scale of 0–63 [35]. In addition to the survey questionnaire, data related to the child and childbirth experience were collected, such as the child’s birth date and whether the child was the first-born [36]. Demographic information such as the mother’s age, family income, and occupation was also gathered. The survey also inquired about how the mothers use social media platforms, like Facebook, to update their thoughts and status.

The PDSS is an online questionnaire that contains a wealth of information specific to the psychometrics of the English version. It has seven components: problems with sleeping and eating, anxiety and insecurity, emotional ability, mental confusion, loss of one’s sense of self, guilt and shame, and thoughts of ending one’s life. Each dimension is composed of five different items, each of which describes another emotion that a woman may be experiencing after the birth of her child. The evaluation was based on the score and confirmed by clinical experts. The participants who scored above a certain threshold were considered affected by postpartum depression and were used for further analysis.

#### Facebook data

Under strict privacy safeguards, participants were requested to grant access to their public Facebook pages before answering questions. All of the information that was accessible through public personal profile pages was gathered through the use of an API from users who scored above a certain threshold on the filled questionnaire. Each participant interaction posts in the group as well as individual thoughts data were collected and analyzed post-collection to predict the presence of PPD.

#### Survey responses

Participants who met the inclusion criteria signed an informed consent form, and their stress levels after delivery were assessed in this study through questionnaires and social media content analysis. The data used to perform this analysis is collected from 496 different profiles. Data collection did not include any personally identifying information that could lead to the identification of individuals. Textual messages, specifically posts after delivery that were written in English, were the focus of the analysis. As a result, text messages written on personal profiles by postpartum depression mothers primarily deal with messages about birth and feelings the mothers face after delivery. It is essential to remember that the data collected from social media platforms contain a significant amount of background noise, and the amount of text produced by each user varies greatly.

### Data cleaning and pre-processing

Data cleaning and pre-processing steps were applied to the dataset to remove any irrelevant or duplicate data and to format the data so the model could quickly analyze. This section describes the procedures for cleaning and processing the dataset prior to the stress detection task. To begin, limits on the minimum required text volume and the total number of posts were imposed to channel the data set. This helped to ensure that only relevant data was used in the analysis and that the sample size was large enough to be statistically meaningful.

#### Description of the cleaning procedures

**Fig. 1:**
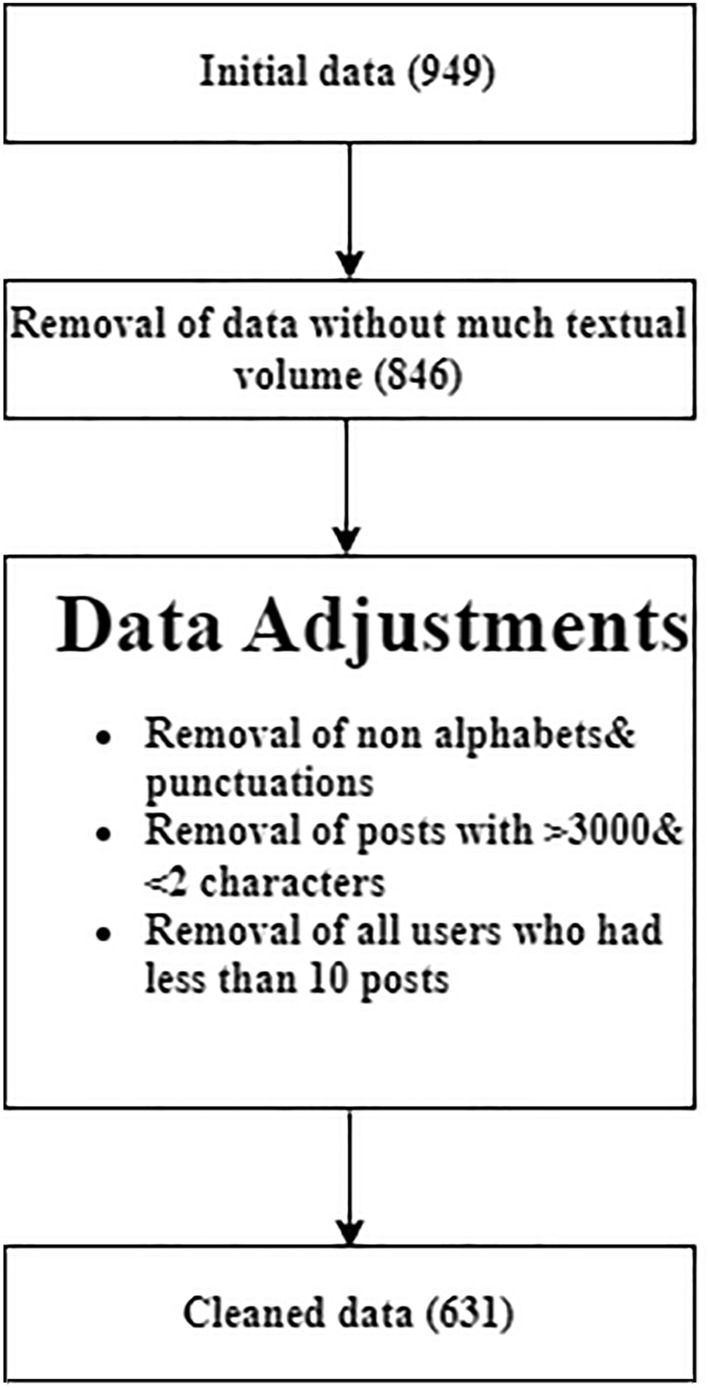
Data cleaning

This paper analyzes the results of the PDSS and classifies mothers into two groups: the control group, whose scores were lower than 11, and the depression group, whose scores were higher than 29. Secondly, the posts were retrieved from these two groups of mothers. The data before cleaning was referred to as the ”initial data,” and the data after the cleaning was referred to as the ”cleaned data.” Figure 1 shows the detailed criteria taken into account for the cleaning process.

**Table 1:** Statistical information regarding the various data preparation phases

Observed data	Initial data	Cleaned data
Number of participants	949	631
Age	24.88 ±6.47	25.99 ±6.11
Depression score	18.97 ±11.68	17.99 ±11.04
Total number of posts	1257	872
Avg. posts count	65.93 ±103.85	61.9 ±29.3
Avg. words count	1314.67 ±1207.82	938.01 ±1044.16
Avg. sentences count	119.96 ±102.78	108.98 ±98.69
Words per post	28.75 ±29.57	22.22 ±14.42
Words per sentence	9.61 ±4.43	8.98 ±2.76
Sentences per post	2.66 ±1.96	2.31 ±0.99

The tables are presented as mean value ± standard deviation

The original data is quite noisy, as illustrated in Table 1. The standard deviation for the post, sentence and word count is doubled compared to their mean values. Additionally, 318 data points from participants lacked textual volume. A superficial examination of the data from the posts revealed that it needs to be adjusted. As the next step, the data was adjusted using regular expressions and removed all random characters that were not alphabet or punctuation marks. Any posts that were either too long (over 3000 characters) or too short (less than two words) were excluded, and also eliminated all users who had less than ten posts. Applying these procedures to the raw data resulted in 631 cleaned user profiles.

**Table 2:** Statistics of participants based on depression annotations score.

Observed data	Depression group	Control group
Number of participants	438 (69.41 %)	193 (30.58 %)
Age	25.67 ± 6.43	25.87 ± 5.21
Depression score	36.44 ± 6.37	6.17 ± 2.75
Total number of posts	358	280
Avg. posts count	87.26 ± 30.13	63.91 ± 29.07
Avg. words count	1028.15 ± 1071.14	908.44 ± 984.7
Avg. sentences count	108.61 ± 113.08	98.54 ± 95.75
Avg. Words per post	22.74 ± 18.58	23.04 ± 13.89

The tables are presented as mean value ± standard deviation

The psychologists involved in the study grouped the data into two groups based on Postpartum Depression Screening Scale (PDSS) and predefined values established by the medical society. Within cleaned data, all mothers with depression annotations scored less than 11 and were categorised as a non-risk group (control group). The depression group was identified as those with scores greater than 29. Individuals who scored between these values and had depression were excluded from observation. The most appropriate method would be using regression analysis with raw depression ratings. Following this method, the number of users in the data population decreased to 314, as shown in Table 2. Of these, 99 were classified as the control group (those who did not exhibit signs of depression), and 215 mothers were classified as belonging to the depression group.

## Methods

**Fig. 2:**
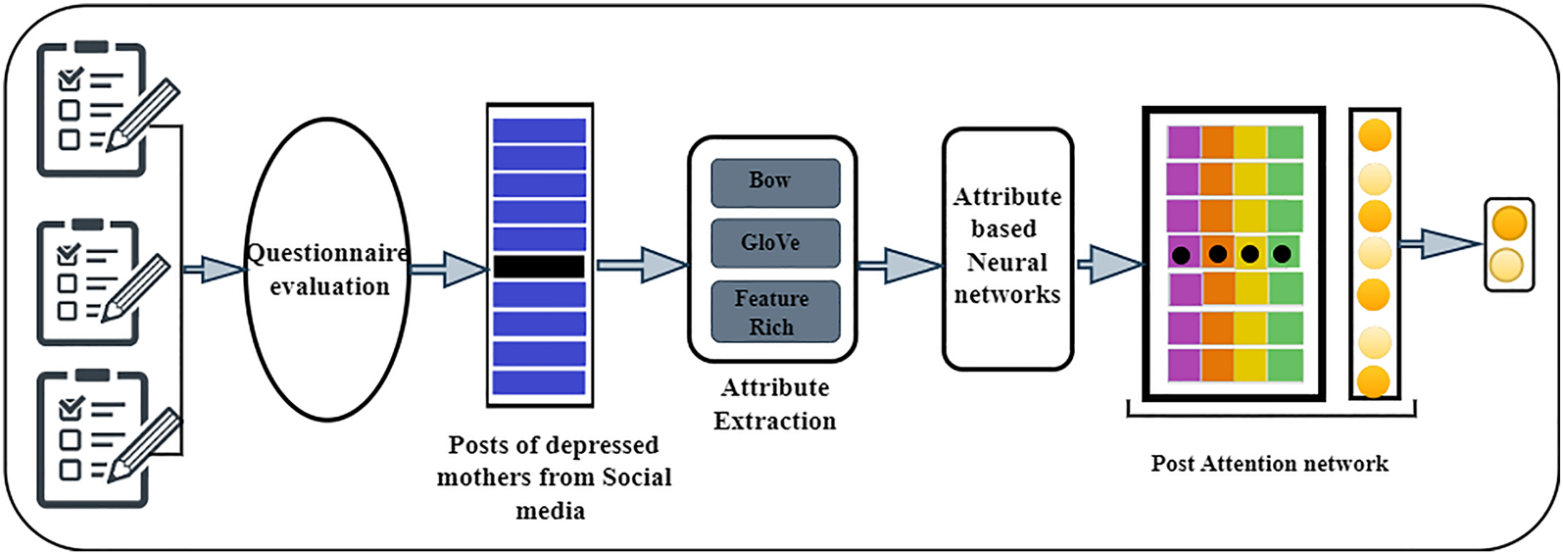
Framework of Attribute Selection Hybrid  Network deep learning models

This section details the findings on depression and the hyperparameters of the utilised model. The Attribute Selection Hybrid Network Models Fig. 2 depicts the entire network architecture that makes up. It consists of two recursive attribute networks based on evaluating the posts and the interconnected networks. Each attribute is executed in accordance with a preexisting depression theory and a post-level attention layer on top of the networks. The process of each network, the post-level attention, is explained in the following parts, the reasoning behind why they are created in such a way and how they are put into practice.

###  Experimental setup

Tables 3, 4 present the hyperparameters used in conjunction with these selected models as similar to [37]. Computing infrastructure used in our research was GEForce GTX 1080 GPU with uniform sampling strategy and training duration of 1234s. The Adam optimizer was used to train all models, and stochastic gradient descent was the training method [38]. In each of the past models, the convolution size, the number of convolutional filters, the pooling type, the pooling length, and the number of dense layers were all comparable.

**Table 3:** Hyper parameter search spaces

Computing Infrastructure	GeForce GTX 1080 GPU
Number of search trails	50
Search strategy	Uniform sampling
Training duration	1482 sec

**Table 4:** The hyper parameters used in the proposed model

Hyperparameter	Search space	Best assignment
Number of epochs	50	50
Batch size	64	64
Gradient norm	Uniform-float [5, 10]	8.0
Embedding dropout	Uniform-float [0, 0.5]	0.3
Number of pre-encode feedforward layers	Choice [1, 2, 3]	3
Number of pre-encode feedforward hidden dims	Uniform-integer [64, 512]	232
Pre-encode feedforward activation	Choice [relu, tanh]	tanh
Pre-encode feedforward dropout	Uniform-float [0, 0.5]	0.0
Encoder hidden size	Uniform-integer [64, 512]	93
Number of encoder layers	Choice [1, 2, 3]	2
Integrator hidden size	Uniform-integer [64, 512]	337
Number of integrator layers	Choice [1, 2, 3]	3
integrator dropout	Uniform-float [0, 0.5]	0.1
Number of output layers	Choice [1, 2, 3]	3
Output hidden size	Uniform-integer [64, 512]	384
Output dropout	Uniform-float [0, 0.5]	0.2
Output pool sizes	Uniform-integer [3, 7]	6
Learning rate optimizer	Adam	Adam
Learning rate	Loguniform-floa t[ 1e−6, 1e−1]	0.0001
Learning rate scheduler	Reduce on plateau	Reduce on plateau
Learning rate scheduler reduction factor	0.5	0.5

### Attribute selection hybrid network models (ASHN)

**Fig. 3:**
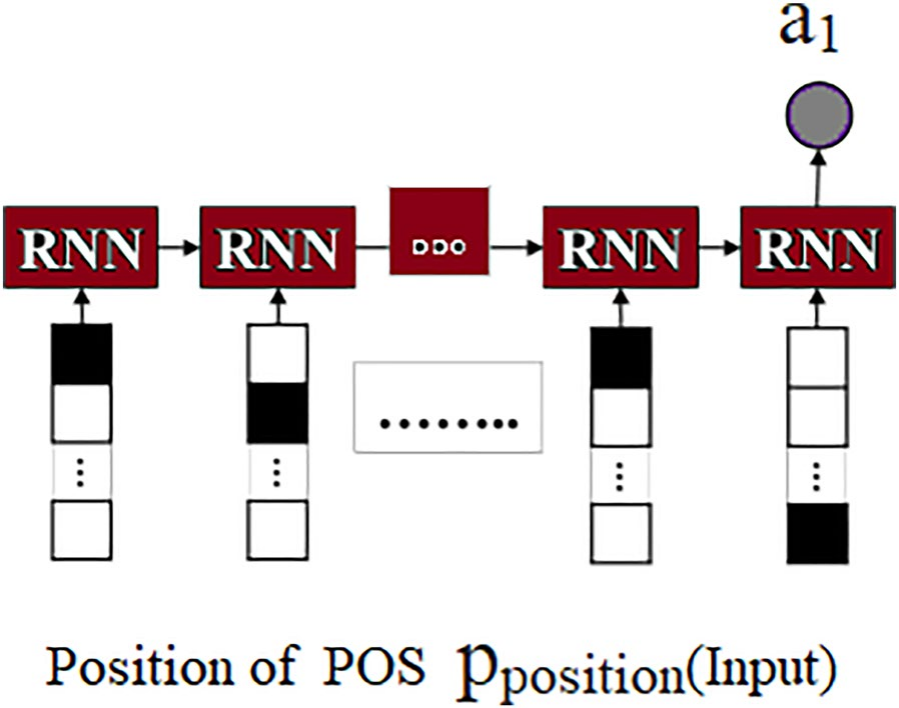
A schema of attribute network to analyze the Psycholinguistic style of the posts by delivered mothers on social media

**Fig. 4:**
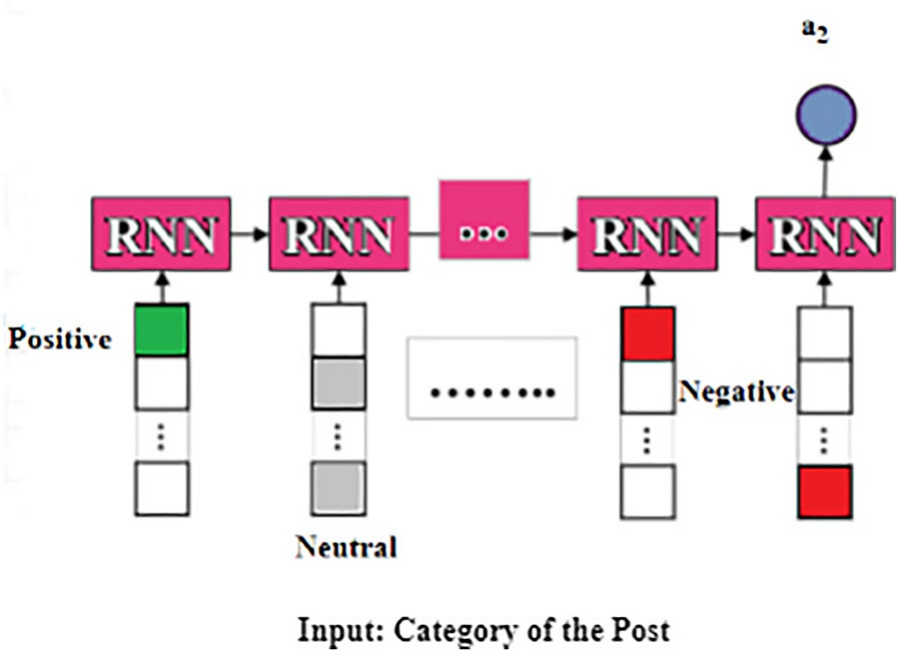
A schema of attribute network for analyzing the sentiments of the posts by delivered mothers on social media such as positive, negative, and neutral

**Fig. 5:**
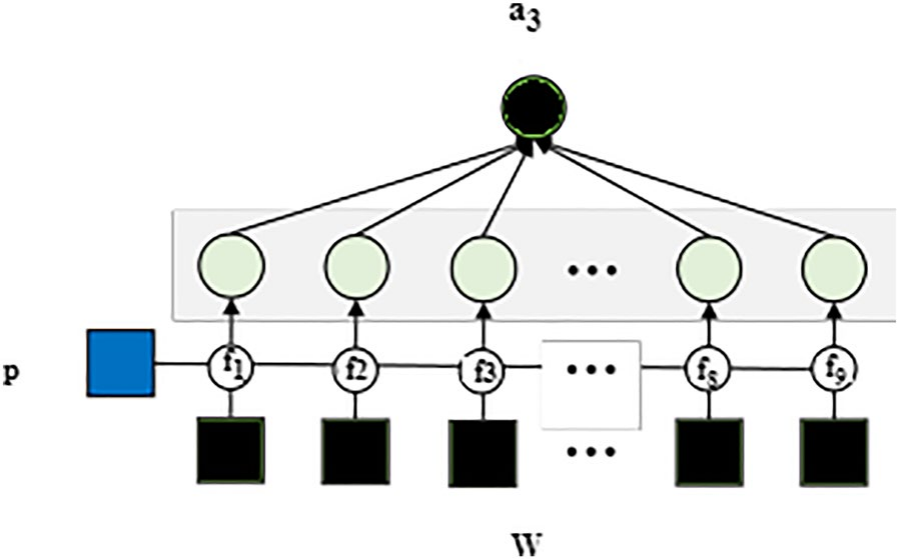
A schema of attribute network to predict the depressive symptoms in the posts posted by delivered mothers on social media

This approach is used by domain experts with expertise in depression to identify depression in mothers’. And their social media post contain positive and negative mood expressions. Here, the positive posts by the depressed mothers were filtered and continued only with the negative posts (296/358) for further analysis, which served as the input of the model for the attribute networks in order to identify the importance of any various attributes in negative posts of the depressed mothers which contribute to predicting the depressed mothers. Fig. 2 shows the Attribute Selection Hybrid Network Models. The attribute networks’ each colourful circles represent a different attribute. Domain expertise is utilized to identify pertinent signs of impending depression. For this reason, four neural networks are created; they are specifically designed for each of the four categories of strong symptoms of depression that are collected from psychological studies. A simple symbol (ex. x) signifies a non-vector in the following explanations. A bold-faced lower-case symbol (ex. x) denotes a vector, while a bold-faced upper-case symbol (ex. X) denotes a collection of vectors or a matrix.

**Fig. 6:**
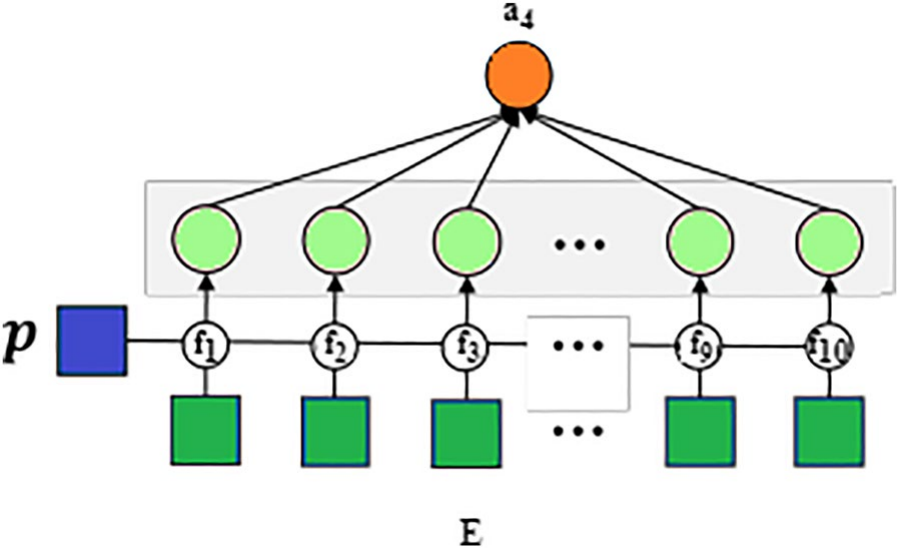
A schema of attribute network for psycholinguistic style

- *Psycholinguistic style (morphological order) (A1)* Some research suggests that people who suffer from depression display distinct linguistic styles, such as differences in the distribution of nouns, verbs, and adverbs and differences in the complexity of their sentences. These linguistic styles are conceptualized unconsciously [39]. The previous study served as the foundation for developing the first type of attribute network, which aimed to recognize various writing styles. In addition to focusing on the multiple styles, attention is given to the order of the words and the distribution of parts- of-speech tags. Consequently, we send a post to the network that consists of a series of parts-of-speech tags. After that, the network will change the sequence into a one-hot vector with the same number of part-of-speech dimensions as the sequence, and it will use RNN to encode the one-hot vector into an attribute vector called $a_1$ as shown in Fig. 3.
  1 $$\begin{aligned} \textbf{a}_1=R N N\left( \textbf{x}_{\text{ pos } }\right) \end{aligned}$$
- *Sentimental words (A2)* The cognitive theory proposes that those who suffer from depression are more likely to exhibit negative thought patterns and negative feelings. As a result, there exists a hypothesis that anyone who is depressed has a greater propensity to express a negative polarity on their postings more frequently than other users on social media. The attribute extraction network is proposed on the above belief that it will identify such behaviour by considering the sentiments expressed in posts as shown in Fig. 4. Towards this end, SentiWordNet made use of computing sentiment scores for each word. By converting all of the words in a post into one of three categories-positive, neutral, and negative-SentiWordNet’s, and then we use a Recurrent Neural Network (RNN) to encode the one-hot vectors into an attribute vector ($a_2$).
  2 $$\begin{aligned} \textbf{a}_2=R N N\left( \textbf{x}_{ \text{ sent }}\right) \end{aligned}$$
- *Depressive symptom words (A3)* It appears that the most distinguishable behavioural pattern of mothers who undergo PPD, posts the comments that are specifically associated with a particular depression symptom. The attribute network shown in Fig. 5 is proposed to find words that are related to depression symptoms in posts, which is based on this discovery. In order to determine which symptom is associated with depression, a dictionary was compiled with evidence keywords using terms taken from the Diagnostic and Statistical Manual of Mental Disorders, Fifth Edition (DSM-V) [40]. This has helped to determine to find out the symptoms that are associated with PPD. The lexicon includes 76 keywords pertaining to nine categories of symptoms described by DSM-V. To compute the similarity between one given post and tokens of the dictionary in order to capture each mothers’ piece of evidence found in posts relating to one of nine symptoms. In the first step of this process, element-wise multiplication was used to combine the word vectors corresponding to each symptom category into a single vector. As a result of this, a symptom matrix was generated that consists of representative vectors for each category. The matrix displays the degree of similarity between an encoding vector of posts and the matrix. In the final step, the Multi-Layer Perceptron(MLP) was used to project the matrix onto the attribute vector ($a_3$).
  3 $$\begin{aligned}{}&\textbf{f}_i=\textbf{x} \textbf{W} \textbf{E}_i \quad (i=1, \ldots , 9) \\&\textbf{Y}={\text {softmax}}\left( \left[ \textbf{f}_1, \textbf{f}_2, \ldots , \textbf{f}_{9}\right] \right) \\&\textbf{a}_3=\tanh (f(\textbf{Y})) \end{aligned}$$
- *Ruminative response style (A4)* It is common knowledge that the ruminative reaction style manifests itself in the form of repetitious behaviours and thoughts. People who suffer from depression have a pattern of continuously expressing their sentiments or dwelling on unfavourable situations, which might lead to sentences on relevant topics repeatedly appearing on their online posts. On the basis of this theory, putting into practice a network that identifies the frequency with which particular stories concerning pertinent issues are repeated is shown in Fig. 6. Computation of two vectors using dot production to determine the degree of relevance between a specific post and others. The degree of significance for each post was derived using this information. After that, MLP was used to convert the degree into an attribute vector designated as $a_4$.
  4 $$\begin{aligned} \textbf{f}&={\text {softmax}}(\textbf{x} \cdot \textbf{E}) \\ \textbf{a}_4&=\tanh (f(\textbf{a}))\end{aligned}$$

Each post demonstrates a unique level of depressive traits; it is vital to take into consideration the weights of the attributes before integrating the attribute networks. As a result, to classify the user based on the analysis of the post, we produce a vector with weights that indicate which attribute is the most representative. The next step is to multiply the weights of the attribute networks. After that, we build a post vector that considers all of the attributes by combining the weighted attribute vector with a vector based on the summation of the elements.

5 $$\begin{aligned} \textbf{w}&={\text {softmax}}(f(\textbf{x})) \\ \textbf{p}^{\prime }&=\sum _i \textbf{w}_i \cdot \textbf{f}_i \quad (i=0, \ldots , 4) \end{aligned}$$

The weights indicate the contribution of each attribute in classifying the post, which helps to explain how and why depression develops, this interpretation of the behaviour was carried out by changing the weights.

### Post-level attention

Even someone who struggles with depression may not always convey their depressed emotions through the postings they make on social media. Due to this reason, the preliminary phase consists of a questionnaire analysis. The results were utilised in conjunction with the forecasts of the medical experts. Moreover, the posts of the individuals on whom the questionnaire focused will not reflect depressive traits in all of the posts. As a result, it is essential to carefully choose and process such postings in accordance with the importance of their respective roles. Similar to the hierarchical attention method [41], the attention mechanism was applied to the posts. To calculate the importance of the postings, a context vector (*v*) was introduced with a post vector(*p*’).

6 $$\begin{aligned} &\textbf{a}={\text {softmax}}\left( \textbf{p}^{\prime } \textbf{W} \textbf{v}\right) \\&\textbf{o}=\sum _i \textbf{a}_i \cdot \textbf{p}_i^{\prime } \quad (i=1, \ldots , M) \end{aligned}$$

where M is the number of posts. o is the output vector for classifying depression using MLP.

### Metrics

In this study, two neural network-based embedding models were compared regarding positive measures (F-measure, recall, True Positive, accuracy, precision) and negative measures (True Negative, False Positive, False Negative). The precision and recall of a model, in addition to its F1 score, are the metrics used to evaluate its accuracy. The percentage of times that a model either accurately or inaccurately predicts a class can be broken down into the following four categories:

- True positives are results in which the model successfully predicts the presence of positive depression symptoms.
- True negatives are results in which the model successfully predicts the absence of depression symptoms.
- If a model incorrectly predicts the presence of the positive depression symptoms (positive class), the result is known as a false positive.
- False negatives are an outcome that occurs when the model predicts (absence of the depression symptoms) the negative class in an incorrect manner.

The recall is the measure that shows the accuracy of the model is in identifying true positives, while precision is the ratio between true positives and all positives.

7 $$\begin{aligned} Recall&=\frac{True Positive}{(Ture Positive +False Negative)} \end{aligned}$$

8 $$\begin{aligned} Precision&=\frac{True Positive}{(Ture Positive +False Positive)} \end{aligned}$$

9 $$\begin{aligned} F_1 Score&= 2 * \frac{(Precision *Recall)}{(Precision +Recall)} \end{aligned}$$

AUC stands for ”Area under the ROC Curve.” AUC provides an aggregate measure of performance across all possible classification thresholds. ROC stands for Receiver Operating Characteristic) Curve. ROC curve measures the performance of a classification model by plotting the rate of true positives against false positives.

GloVe [42] is used to embed word vectors, and GRU [43], an RNN variant, is used to encode the sequence. Dropout and L2 regularization are used to improve generalization. We have chosen 0.001 for the learning rate and 0.0001 for the L2 regularization rate. We set a dropout rate for each model separately, with 0.3 and 0.2 for the baseline and our model, respectively.The words that appeared more than five times in the vocabulary are kept, and others are replaced the rest with UNK tokens.

## Results

In order to choose the posts for analysis, various metrics were considered, such as analyzing the title of posts and the content of the posts. The titles and contents of the posts were visualized using a word cloud to determine the posts related to PDD as similar to [44]. To identify PDD-related posts and anticipate the most regularly used terms, the most frequently used words in the titles of posts in each category were visualized.

**Fig. 7:**
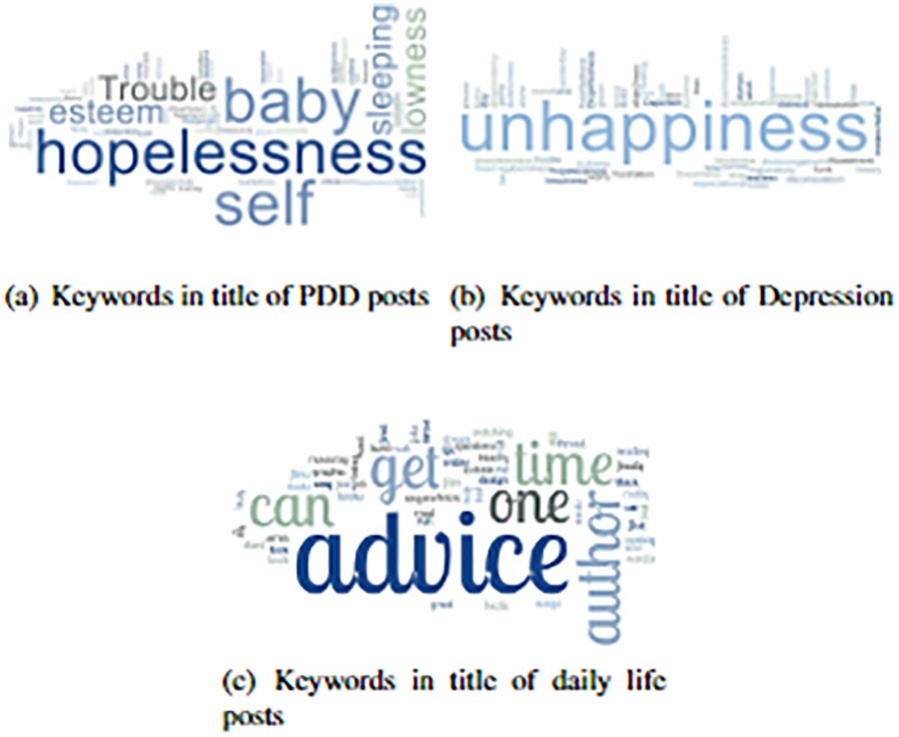
Word clouds for the titles of posts PPD, depression and daily life

**Fig. 8:**
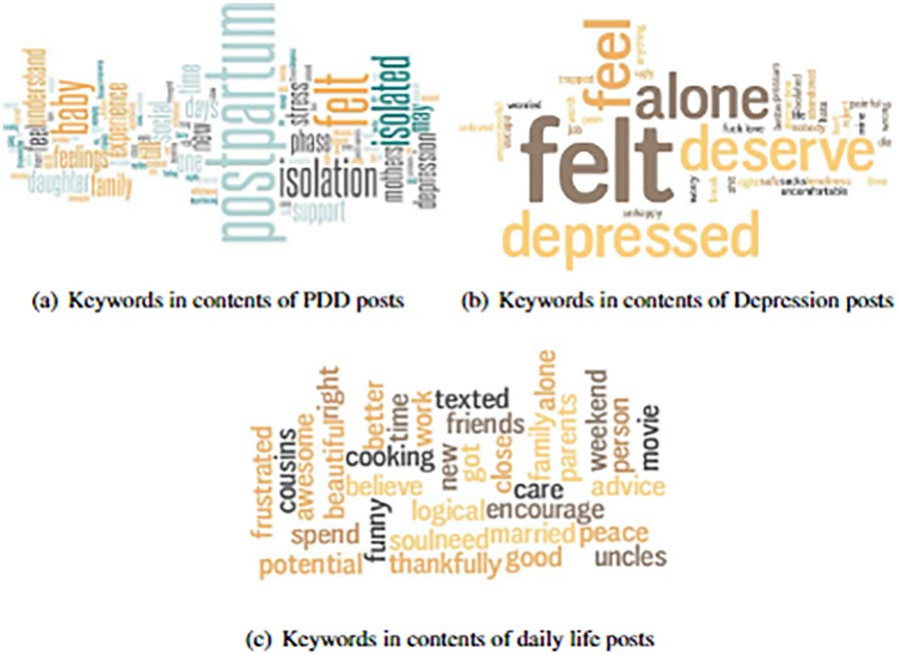
Word clouds for the contents of posts PPD, depression and daily life

The word clouds plotted for the title and content of each post category were depicted in Figs. 7, and 8. Despite the variations in the keyword used to retrieve the posts of title and content in daily life, Fig. 7a,b and has a significantly distinct set of terms than the other two categories; it can be seen that Figs. 7, 8a and 7, 8b have numerous term occurrences. There is an evident variation in the frequency of usage, even among phrases that appear in both the PPD and depression categories. Furthermore, specific frequently occurring words, such as baby, PPD, and birth, were only found in the PPD group, which makes sense considering that PPD relates to parents and parenting journeys. Overlap in word usage in the title and content can be seen by comparing corresponding word clouds, as shown in Figs. 7 , 8, which share several terms that were used to crosscheck the content vector calculations in post level attention framework.

Each post was split into a sequence of tokens and performed part-of-speech tagging using Stanford CoreNLP [45]. Posts whose number of tokens was either smaller than five or bigger than 100 were discarded. Then about 245 posts were randomly selected from the whole posts for each user and used for training. The neural models encode posts to vectors using a convolutional neural network (CNN) and then merge the post vectors into a single vector. To distinguish between the conventional network and the attribute network based on human intuition, a baseline model was created by replacing the ASHN attribute network with a bidirectional RNN.

In order to complete the assignment, we have used the scikit-learn machine learning library [46]. The examination of the data is carried out with the assistance of the Multinomial Naive Bayes(MNB) and Support-Vector Machines (SVM) models. Normalization and scaling were applied to each and every attribute set. Grid-search iterations are utilized to fine-tune the hyperparameters of the classification algorithms. The results of diagnosing depressed users on the collected test set are presented in Table 5.

**Table 5:** Results of evaluation on the test set

Method	Precision	Recall	Accuracy	F1 score
BOW^a^	51.68±9.89	52.17±3.70	64.59±3.79	53.84±6.35
BOW^a^	55.43±1.99	59.82±1.88	61.12±4.46	62.92±1.51
GloVe^a^	58.21±5.52	56.30±7.20	58.07±6.33	50.66±5.80
GloVe^b^	60.64±6.67	62.47±6.06	63.18±2.68	60.12±3.16
Feature rich^a^	59.80±6.21	59.80±6.21	70.91±6.81	54.47±3.66
Feature rich^b^	**62.60**±**7.77**	60.26±7.88	74.89±4.05	61.59±2.20
Baseline	70.78±6.07	**73.74**±**11.24**	69.29±2.64	75.12±2.76
ASHN	**78.77**±**2.99**	72.94±1.88	**74.77**±**3.24**	**75.74**±**1.33**

^a^ and ^b^indicate MNB and SVM classifiers, respectively

Bolder values indicate the better results than other attribute selection methods

Table 5 reveals two important characteristics of the ASHN, based on the post-level attention weights and change in the effect of posts with high attention.

- ASHN and our baseline model have similar F1 scores, but their performance balance differs. Even though ASHN has  more precision than recall, the trend in the baseline is the opposite. To figure out why we have analyzed the post-level attention weights from both models to find important factors and interpret ways that are used to group depressed users. We chose the top 100 posts (20%) from each of the nearly 215 depressed users labelled as depressed by both models. These posts had the highest attention weights for each model. We have found that, on average, only 46 baseline and FAN posts are the same. This means that when two models come up with different attention weights, it usually leads to different results and performance when detecting posts.
- In addition, to analyze the change in the effect of posts with high attention produced by the two models, we present the attention weights of the top 100 posts, averaging nearly 215 users. Interestingly, the baseline’s highest attention weight is marginally more significant than ASHN. It is interesting that the baseline’s highest attention weight is a little more prominent than ASHN’s. Based on this, the baseline classifies users based on a small number of posts with high attention weights, while ASHN classifies users based on a large number of posts more evenly. This means that if only a small number of posts are messed up, there is a higher chance of baseline inaccuracy. On the other hand, the classification is based on a more significant number of posts, and the results produced by the ASHN are trust-able. This explains why the two models have different scores for accuracy and recall.

## Discussion

By looking at the learned representations, we use ASHN to figure out what the detection results mean. We chose a group of almost 290 depressed mothers whose depression symptoms were found to be confirmed. Then, for each mother, we took a sample of the top 100 posts with most attention and the bottom 100 posts with the least attention. We also chose a group of almost 120 depressed mothers who were found to be false-negatives. For each of these users, we picked 100 of their best and worst posts similarly. The average feature weights for each of the four classes are shown in Table 6. The Table 6 below displays the typical amount of attention paid to each attribute( four classes) in the posts.

**Table 6:** True-positive and true-negative values of each attribute based on high and low attention

	Attentions			
Attributes	High	Low	High	Low
Psycholinguistic (morphological) A1	0.33	0.86	0.42	0.84
Ruminative Response style A2	0.13	0.08	0.19	0.09
Sentimental words A3	**0.63**	0.06	0.24	0.08
Depressive symptom words A4	0.33	**0.85**	0.46	**0.86**
	**True positive** **(TP)**		**True negative** **(TN)**	

Bolder values indicate the importance of the attributes to predict the depression in PDD posts

Examining the Table 6 instances for each class to ensure ASHN provides sufficient results to meet the objectives.

- *A1:Psycholinguistic style *The morphological writing style of a post, also known as A1, has a relatively minor impact on the ability to detect depression compared to other attribute networks. Every word in this post has been assigned a tag that corresponds to a component of the usage of words, which explains the purpose of each word. Tags for different parts of speech are determined by the connections between the individual words that make up the phrase. Models based on machine learning are used to determine the parts of speech tags associated with a word. The Penn Treebank corpus offers the tag notations that are utilized the majority of the time for the various elements of speech. Wherein a total of 48 (Parts Of Speech) P.O.S tags are defined in accordance with their respective applications. On the other hand, research has shown that an increase in the A1 weight also results in an increase in the number of verb phrases. When compared to the various forms of nouns, Table 7 demonstrates that an increase in the frequency of verbs results in a proportional rise in attention. This seems to imply that mothers who have issues with their mental health display a distinct level of sentence complexity when it comes to their language [39].
- *A2:Sentimental words* Regarding the second attribute weight (A2), it is discovered that the higher A2 weight (0.63) displays, higher the attention scores in the posts. This implies that sentiment information is important in detecting depressed users. Table 8 displays the most common words and their polarity in a group of post with high A2 weights. The word ’hopeless,’ which has a negative polarity, for example, does not appear in the group of low A2 weighted posts from users in the TP-High and TP-Low classes. In contrast, it appears 978 in terms of the second attribute weight (A2); it has been discovered that the higher the post, displays higher attention scores the post. This implies that sentimental information is important in detecting depressed users. Table 8 displays the most common words and their polarity in a group of posts with high A2 weights. The word ’hopeless,’ which has a negative polarity, does not appear in the group of low A2 weighed posts from users in the TP-High and TP-Low classes, for example. In contrast, ’panic’ appears 236 times in 872 posts in the high A2 weighed posts group, implying that 13.8% of the high A2 weighted posts contain this word. When compared to the most common general words, such as ’baby’(723 times, 74.8%), like’ (752 times, 56%), and ’would’ (511 times, 41.8%), the frequency of ’hopeless’ in this group of posts is relatively high. Furthermore, the Table 8 shows that the majority of the top-ranked frequent words in the high A2 weighted posts have negative polarity, resulting in a post with a negative and depressive mood. This means that in detecting depression in Primi-depressed mothers, the attribute network based on sentiment information significantly distinguishes depressive posts from Non-Primi mothers.
- *A3: Depressive symptom words* Posts with high A3 weights are mostly found in the TP-Low category, as shown in Table 6. Furthermore, it is discovered that the words in Table 8 frequently appear in the low A3 weighed posts showing the reason for depressive symptoms is negatively associated with attention. Most posts do not appear to be related to depressive mentions if the A3 weight of the post is low because the keywords selected for depressive symptoms rarely appear throughout the entire post. In other words, a post does not relate to any of the attributes, identified that it shows biased attribute weights toward A3.
- *A4: Ruminative Response Style* Some posts in the TP-High class, however, display high A3 and A4 weights. Examples of phrases from them are shown in Table 9. Many posts containing these phrases are tied to the practice of so-called ”self-attention,” in which users regularly discuss their feelings or experiences. It is analyzes the frequency of the word ”I” in all posts from the two classes of posts (TP-High and TP-Low), as well as in specific posts with A3 weights higher than 0.50 and A4 weights higher than 0.15, in order to analyze this pattern further. ’I’ appear on average 1.25 times across all posts and 1.35 times across high A3 and A3 weighted posts. In the TP-High class, the percentage of postings with high A3 and A4 weights$\ (A3> 0.43, A4 > 0.14)$ is 14.8%, compared to 0.4% in the TP-Low class. This demonstrates that individuals with mental health issues have a high level of self-awareness [47].

**Table 7:** Polarity for various parts of speech tags

Tag	Explanation	TP-High	TP-Low
NN	Noun, singular	0.32	0.38
NNS	Noun, plural(Non-singular)	0.21	0.28
NNP	Proper noun, singular	0.43	0.57
NNPS	Proper noun, non singular (Plural)	0.25	0.36
VB	Verb, base form	1.83	1.67
VBD	Verb, past tense	1.04	0.95
VBG	Verb, gerund/ past participle	0.83	0.76
BN	Verb, past participle	0.59	0.54
VBP	Verb, non-3rd ps,sing,present	1.76	1.61
VBZ	verb, 3rd ps, sing, present	1.18	1.08

**Table 8:** A collection of frequent words in PDD mothers’ posts and their polarity that determined using SentiWordNet

Words	Frequency	Polaritiy
Hopeless	978	Negative
Sleepless	937	Negative
Tired	872	Negative
Hurting	923	Negative
Anxious	821	Negative
Overeating	723	Negative
Panic	236	Negative
Crying	176	Negative
Medication	142	Positive
Planning	56	Positive

**Table 9:** Phrases from posts with high A3 and A4 weights as examples

A3	A4	Phrase
0.43	0.19	I feel like
0.41	0.19	I am helpless
0.36	0.16	I am restless
0.32	0.14	I am staying asleep

### Limitation and future work

However, due to limited computational capacity, as mentioned in Sect. 4.1, our model uses less training data as input, resulting in inferior performance than the leading model, which uses three times as much data as ours and is trained in a less interpretable manner. It is believed that as computational power increases, our model has the ability to outperform the state-of-the-art model.In this paper, only binary classification (depressed or non-depressed) is considered. Phycological Scores of people less than 11 and grater than 29 are considered. ASHN now consists of only four aspects based on depressive psychiatric studies. It is clear evidence that to enhance performance, no samples were gathered for analysis, the number of attributes taken for analysis, and the computing power of the model used plays a predominant role. Given the widespread adoption of these technologies, there is now a window of opportunity to observe and analyze long-term user behaviour patterns among mothers after giving birth. This can provide valuable insight into the attributes contributing to PPD and help develop better early identification and treatment methods. Because our model employs high-dimensional representations of neural networks by allowing the incorporation of other high-level attributes and adding other valuable attributes to the model. It will enable us to generate more reasonable and diverse explanations for many elements of depression. We recreate the diagnosis process similarly if we can construct adequate attribute networks for other mental disorders (such as dementia, schizophrenia, and bipolar disorder). Moreover we can also extend into multi classification with individuals scored between 11 and 29 trapped with mild, moderate, serve, etc., cases.

## Conclusions

This research has developed deep learning methods to improve PPD detection even more than the traditional labor-intensive approaches that use manual intervention for attribute collection. The suggested Attribute Selection Hybrid Network (ASHN) mimics the process of detecting depressed mothers through their social media posts. The input of our given models are posts gathered from PPD-depressed women, who were concluded as depressed by answering a questionnaire designed by domain experts. The attributes chosen for predicting PPD, such as Psycholinguistic style, sentimental words, depressive symptom words and Ruminative Response Style, are based on the advice of the domain experts. Furthermore, this model focuses more on attributes of depression-related sentences which matches well with the real-world situation in which only a few posts are relevant to depression, even for depressed mothers. Thus, ASHN uses a post attention mechanism to choose carefully and posts according to the importance of their respective roles based on considering context vectors. It also enables interpretation of why a particular post is connected to depression in terms of psychological study aspects by analyzing the keywords used in the PPD as general depression as depicted in visualization using word cloud, which is helpful for subsequent clinical investigation of depressive symptoms.

## Data Availability

On reasonable request and if data transfer agreements are in place, the corresponding author will provide the datasets created and/or analyzed during the current study available to users.

## Abbreviations

ASHN: Attribute Selection Hybrid Network
AUC: Area Under Curve
BOW: Bag Of Words
CNN: Convolutional Neural Network
DSM-V: Diagnostic and Statistical manual of Mental disorders, fifth edition
FP: False Positive
FN: False Negative
GloVe: Global Vectors
GRU: Gated Recurrent Unit
IEC: Institutional Ethical Committee
MLP: Multi-Layer Perceptron
MNB: Multinomial Naive Bayes
PPD: Postpartum Depression
PDSS: Postpartum Depression Screening Scale
P.O.S: Parts Of Speech
RNN: Recurrent Neural Network
ROC: Receiver Operating characteristic) Curve
SVM: SupportVector Machines
SRMC & RC[MYAMPRC]: SRM Medical college and Research Centre
TP: True Positive
TF: Ture Negative
